# Diaqua­bis(5-carb­oxy-2-propyl-1*H*-imidazole-4-carboxyl­ato-κ^2^ *N* ^3^,*O* ^4^)nickel(II) *N*,*N*-dimethyl­formamide disolvate

**DOI:** 10.1107/S1600536810004691

**Published:** 2010-02-13

**Authors:** Shi-Jie Li, Jian-Bin Yan, Wen-Dong Song, Hao Wang, Dong-Liang Miao

**Affiliations:** a College of Food Science and Technology, Guang Dong Ocean University, Zhanjiang 524088, People’s Republic of China; b College of Science, Guang Dong Ocean University, Zhanjiang 524088, People’s Republic of China

## Abstract

In the title complex, [Ni(C_8_H_9_N_2_O_4_)_2_(H_2_O)_2_]·2C_3_H_7_NO, the Ni^II^ atom is six-coordinated by two *N*,*O*-bidentate 5-carb­oxy-2-propyl-1*H*-imidazole-4-carboxyl­ate ligands and two water mol­ecules in a distorted octa­hedral environment. The methyl C and H atoms of the two ligands are disordered over two sets of sites in 0.74 (2):0.26 (2) and 0.57 (8):0.43 (8) ratios. A supra­molecular network is stabilized by intra- and inter­molecular N—H⋯O and O—H⋯O hydrogen bonds involving the ligands, coordinated water mol­ecules and dimethyl­formamide solvent mol­ecules.

## Related literature

For the structures of related 2-propyl-1*H*-imidazole-4,5-dicarboxyl­ate complexes, see: Song *et al.* (2010 ▶); Yan *et al.* (2010 ▶).
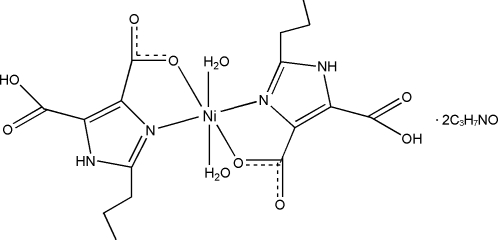


## Experimental

### 

#### Crystal data

[Ni(C_8_H_9_N_2_O_4_)_2_(H_2_O)_2_]·2C_3_H_7_NO
*M* *_r_* = 635.26
Orthorhombic, 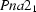

*a* = 16.3574 (12) Å
*b* = 9.5246 (7) Å
*c* = 18.7700 (13) Å
*V* = 2924.3 (4) Å^3^
*Z* = 4
Mo *K*α radiation
μ = 0.73 mm^−1^
*T* = 273 K
0.31 × 0.24 × 0.18 mm

#### Data collection

Rigaku/MSC Mercury CCD diffractometer
Absorption correction: multi-scan (*REQAB*; Jacobson, 1998 ▶) *T*_min_ = 0.805, *T*_max_ = 0.880
14413 measured reflections
5064 independent reflections
3663 reflections with *I* > 2σ(*I*)
*R*_int_ = 0.063

#### Refinement

*R*[*F* ^2^ > 2σ(*F* ^2^)] = 0.048
*wR*(*F* ^2^) = 0.133
*S* = 1.03
5064 reflections
403 parameters
1 restraint
H atoms treated by a mixture of independent and constrained refinement
Δρ_max_ = 0.37 e Å^−3^
Δρ_min_ = −0.36 e Å^−3^
Absolute structure: Flack (1983 ▶), 2344 Friedel pairs
Flack parameter: 0.01 (2)

### 

Data collection: *CrystalStructure* (Rigaku/MSC, 2002 ▶); cell refinement: *CrystalStructure*; data reduction: *CrystalStructure*; program(s) used to solve structure: *SHELXS97* (Sheldrick, 2008 ▶); program(s) used to refine structure: *SHELXL97* (Sheldrick, 2008 ▶); molecular graphics: *ORTEPII* (Johnson, 1976 ▶) and *DIAMOND* (Brandenburg, 1999 ▶); software used to prepare material for publication: *SHELXL97*.

## Supplementary Material

Crystal structure: contains datablocks I, 1R. DOI: 10.1107/S1600536810004691/hy2277sup1.cif

Structure factors: contains datablocks I. DOI: 10.1107/S1600536810004691/hy2277Isup2.hkl

Additional supplementary materials:  crystallographic information; 3D view; checkCIF report

## Figures and Tables

**Table 1:** Hydrogen-bond geometry (Å, °)

*D*—H⋯*A*	*D*—H	H⋯*A*	*D*⋯*A*	*D*—H⋯*A*
O3—H1⋯O2	1.05 (6)	1.42 (6)	2.474 (6)	176 (5)
O7—H7⋯O6	0.82	1.67	2.479 (6)	169
N2—H2⋯O10^i^	0.86	1.93	2.780 (6)	171
N4—H4⋯O9	0.86	1.94	2.788 (6)	171
O1*W*—H1*W*⋯O8^ii^	0.85	1.96	2.782 (5)	162
O1*W*—H2*W*⋯O10^iii^	0.85	1.92	2.757 (6)	168
O2*W*—H3*W*⋯O4^iv^	0.85	1.96	2.794 (5)	166
O2*W*—H4*W*⋯O9^v^	0.85	1.98	2.800 (6)	163

Symmetry codes: (i) 
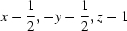
; (ii) 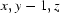; (iii) 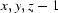; (iv) 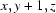; (v) 
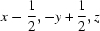
.

**Fig. 1.**
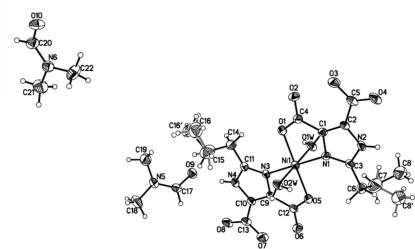
Molecular structure of the title compound, showing the 30% probability displacement ellipsoids.

**Fig. 2.**
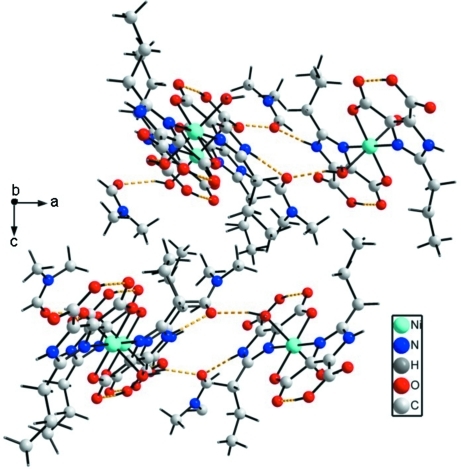
A view of the two-dimensional network constructed by N—H···O and O—H···O hydrogen bonding interactions (dashed lines).

## supplementary crystallographic information

### Comment

2-Propyl-1*H*-imidazole-4,5-dicarboxylate ligand with efficient N,*O*-donors has been used to obtain new metal–organic complexes, such as poly[diaquabis(5-carboxy-2-propyl-1*H*-imidazole-4-carboxylato- κ^3^*N*^3^,*O*^4^,*O*^5^)calcium(II)] (Song *et al.*, 2010) and [diaquabis(5-carboxy-2-propyl-1*H*-imidazole-4-carboxylato- κ^2^*N*^3^,*O*^4^)manganese(II)] *N*,*N*-dimethylformamide (Yan *et al.*, 2010). In this paper, we report the synthesis and structure of a new nickel(II) complex obtained under hydrothermal conditions.

As illustrated in Fig. 1, the title complex molecule is composed of one Ni^II^ ion, two mono-deprotonated 2-propyl-1*H*-imidazole-4,5-dicarboxylate ligands, two coordinated water molecules and two dimethylformamide solvent molecules. The Ni^II^ atom exhibits a slightly distorted octahedral coordination geometry, defined by two N,*O*-bidentate ligands and two water molecules. In the crystal structure, the complex molecules and dimethylformamide solvent molecules are linked by N—H···O and O—H···O hydrogen bonds (Table 1) into a two-dimensional supramolecular structure parallel to (0 0 1) (Fig. 2). The methyl C and H atoms of the two ligands are disordered over two sites in raios of 0.74 (2):0.26 (2) and 0.57 (8):0.43 (8).

### Experimental

A mixture of Ni(CH_3_CO_2_)_2_ (0.5 mmol, 0.06 g) and 2-propyl-1*H*-imidazole-4,5-dicarboxylic acid (0.5 mmol, 0.99 g) in 15 ml of dimethylformamide solution was sealed in an autoclave equipped with a Teflon liner (20 ml) and then heated at 433 K for 4 d. Crystals of the title compound were obtained by slow evaporation of the solvent at room temperature.

### Refinement

C- and N-bound H atoms were placed at calculated positions and treated as riding on the parent atoms, with C—H = 0.93 (CH), 0.97 (CH_2_) and 0.96 (CH_3_) Å and N—H = 0.86 Å and with *U*_iso_(H) = 1.2(1.5 for methyl)*U*_eq_(C,*N*). The water H atoms were located in a difference map and refined as riding, with O—H = 0.85 Å and *U*_iso_(H) = 1.5*U*_eq_(O). H atoms of carboxyl groups were located in a difference map, and one H atom (H3) was refined isotropically and the other (H7) was refined as riding with O—H = 0.82 Å and *U*_iso_(H) = 1.5*U*_eq_(O).

### Crystal data

**Table:**

[Ni(C_8_H_9_N_2_O_4_)_2_(H_2_O)_2_]·2C_3_H_7_NO	*F*(000) = 1336
*M**_r_* = 635.26	*D*_x_ = 1.443 Mg m^−^^3^
Orthorhombic, *P**n**a*2_1_	Mo *K*α radiation, λ = 0.71073 Å
Hall symbol: P 2c -2n	Cell parameters from 3420 reflections
*a* = 16.3574 (12) Å	θ = 3.3–27.4°
*b* = 9.5246 (7) Å	µ = 0.73 mm^−^^1^
*c* = 18.7700 (13) Å	*T* = 273 K
*V* = 2924.3 (4) Å^3^	Block, green
*Z* = 4	0.31 × 0.24 × 0.18 mm

### Data collection

**Table:**

Rigaku/MSC Mercury CCD diffractometer	5064 independent reflections
Radiation source: fine-focus sealed tube	3663 reflections with *I* > 2σ(*I*)
graphite	*R*_int_ = 0.063
ω scans	θ_max_ = 25.2°, θ_min_ = 2.2°
Absorption correction: multi-scan (REQAB; Jacobson, 1998)	*h* = −15→19
*T*_min_ = 0.805, *T*_max_ = 0.880	*k* = −10→11
14413 measured reflections	*l* = −22→22

### Refinement

**Table:**

Refinement on *F*^2^	Secondary atom site location: difference Fourier map
Least-squares matrix: full	Hydrogen site location: inferred from neighbouring sites
*R*[*F*^2^ > 2σ(*F*^2^)] = 0.048	H atoms treated by a mixture of independent and constrained refinement
*wR*(*F*^2^) = 0.133	*w* = 1/[σ^2^(*F*_o_^2^) + (0.0549*P*)^2^ + 0.3624*P*] where *P* = (*F*_o_^2^ + 2*F*_c_^2^)/3
*S* = 1.03	(Δ/σ)_max_ = 0.001
5064 reflections	Δρ_max_ = 0.37 e Å^−^^3^
403 parameters	Δρ_min_ = −0.36 e Å^−^^3^
1 restraint	Absolute structure: Flack (1983), 2344 Friedel pairs
Primary atom site location: structure-invariant direct methods	Flack parameter: 0.01 (2)

### Fractional atomic coordinates and isotropic or equivalent isotropic displacement parameters (Å^2^)

**Table:**

	*x*	*y*	*z*	*U*_iso_*/*U*_eq_	Occ. (<1)
Ni1	0.10751 (4)	0.00168 (7)	0.09532 (6)	0.03620 (17)	
N1	0.0395 (3)	−0.1796 (4)	0.0878 (2)	0.0372 (10)	
N2	−0.0320 (3)	−0.3724 (4)	0.0753 (2)	0.0406 (12)	
H2	−0.0680	−0.4303	0.0594	0.049*	
N3	0.1752 (3)	0.1835 (4)	0.1021 (2)	0.0365 (9)	
N4	0.2477 (3)	0.3758 (4)	0.1143 (2)	0.0390 (12)	
H4	0.2833	0.4340	0.1306	0.047*	
N5	0.3912 (3)	0.7166 (5)	0.2536 (3)	0.0498 (12)	
N6	0.3277 (3)	0.2034 (5)	0.9330 (3)	0.0501 (12)	
O1	0.1630 (2)	−0.1165 (3)	0.1768 (2)	0.0469 (10)	
O2	0.1603 (3)	−0.3285 (4)	0.2279 (2)	0.0554 (11)	
O3	0.0825 (3)	−0.5493 (4)	0.2133 (2)	0.0573 (11)	
O4	−0.0164 (3)	−0.6333 (4)	0.1436 (3)	0.0591 (12)	
O5	0.0526 (2)	0.1182 (3)	0.0127 (2)	0.0468 (10)	
O6	0.0577 (3)	0.3260 (4)	−0.0417 (2)	0.0531 (10)	
O7	0.1355 (3)	0.5476 (5)	−0.0274 (2)	0.0598 (12)	
H7	0.1149	0.4704	−0.0345	0.090*	
O8	0.2317 (3)	0.6357 (4)	0.0427 (2)	0.0569 (11)	
O9	0.3702 (2)	0.5395 (4)	0.1757 (3)	0.0588 (11)	
O10	0.3442 (2)	0.0345 (4)	1.0158 (3)	0.0612 (12)	
O1W	0.1905 (2)	−0.0835 (4)	0.0234 (2)	0.0487 (10)	
H1W	0.1925	−0.1726	0.0256	0.073*	
H2W	0.2379	−0.0513	0.0145	0.073*	
O2W	0.0247 (2)	0.0860 (4)	0.1677 (2)	0.0487 (10)	
H3W	0.0187	0.1712	0.1553	0.073*	
H4W	−0.0204	0.0436	0.1612	0.073*	
C1	0.0669 (3)	−0.2786 (5)	0.1358 (3)	0.0350 (12)	
C2	0.0221 (3)	−0.3993 (5)	0.1284 (3)	0.0342 (12)	
C3	−0.0202 (3)	−0.2402 (5)	0.0517 (3)	0.0390 (13)	
C4	0.1346 (4)	−0.2387 (6)	0.1836 (3)	0.0416 (13)	
C5	0.0282 (4)	−0.5373 (5)	0.1632 (3)	0.0469 (14)	
C6	−0.0674 (4)	−0.1725 (6)	−0.0067 (3)	0.0556 (16)	
H6A	−0.0729	−0.0731	0.0035	0.067*	
H6B	−0.1218	−0.2126	−0.0081	0.067*	
C7	−0.0265 (6)	−0.1908 (10)	−0.0809 (4)	0.097 (3)	
H7A	−0.0490	−0.1214	−0.1133	0.116*	0.744 (18)
H7B	0.0316	−0.1722	−0.0763	0.116*	0.744 (18)
H7'A	0.0113	−0.1125	−0.0843	0.116*	0.256 (18)
H7'B	0.0076	−0.2734	−0.0753	0.116*	0.256 (18)
C8	−0.0374 (9)	−0.3302 (15)	−0.1122 (7)	0.107 (5)	0.744 (18)
H8A	−0.0056	−0.3974	−0.0859	0.160*	0.744 (18)
H8B	−0.0196	−0.3289	−0.1609	0.160*	0.744 (18)
H8C	−0.0941	−0.3558	−0.1102	0.160*	0.744 (18)
C8'	−0.064 (3)	−0.209 (4)	−0.154 (2)	0.112 (17)	0.256 (18)
H8'1	−0.0230	−0.2402	−0.1867	0.168*	0.256 (18)
H8'2	−0.0862	−0.1212	−0.1697	0.168*	0.256 (18)
H8'3	−0.1070	−0.2778	−0.1514	0.168*	0.256 (18)
C9	0.1496 (3)	0.2789 (5)	0.0522 (3)	0.0341 (12)	
C10	0.1940 (3)	0.4013 (5)	0.0583 (3)	0.0370 (12)	
C11	0.2348 (3)	0.2449 (5)	0.1388 (3)	0.0405 (13)	
C12	0.0820 (4)	0.2391 (5)	0.0046 (3)	0.0397 (12)	
C13	0.1883 (4)	0.5381 (5)	0.0228 (3)	0.0444 (13)	
C14	0.2779 (4)	0.1856 (6)	0.2005 (3)	0.0566 (17)	
H14A	0.3364	0.1909	0.1926	0.068*	
H14B	0.2633	0.0874	0.2057	0.068*	
C15	0.2557 (7)	0.2659 (11)	0.2700 (5)	0.108 (3)	
H15A	0.2523	0.3657	0.2602	0.130*	0.43 (8)
H15B	0.2029	0.2348	0.2872	0.130*	0.43 (8)
H15C	0.2922	0.3459	0.2735	0.130*	0.57 (8)
H15D	0.2006	0.3024	0.2654	0.130*	0.57 (8)
C16	0.320 (4)	0.239 (7)	0.326 (2)	0.110 (14)	0.43 (8)
H16A	0.3189	0.1423	0.3402	0.165*	0.43 (8)
H16B	0.3094	0.2972	0.3672	0.165*	0.43 (8)
H16C	0.3732	0.2616	0.3075	0.165*	0.43 (8)
C16'	0.280 (3)	0.190 (4)	0.3375 (17)	0.109 (10)	0.57 (8)
H16D	0.3303	0.1399	0.3295	0.163*	0.57 (8)
H16E	0.2381	0.1259	0.3510	0.163*	0.57 (8)
H16F	0.2885	0.2577	0.3750	0.163*	0.57 (8)
C17	0.3560 (4)	0.6586 (6)	0.1975 (3)	0.0492 (15)	
H17	0.3177	0.7118	0.1728	0.059*	
C18	0.3694 (6)	0.8590 (7)	0.2739 (4)	0.084 (3)	
H18A	0.3338	0.8985	0.2386	0.125*	
H18B	0.4181	0.9150	0.2774	0.125*	
H18C	0.3421	0.8577	0.3192	0.125*	
C19	0.4482 (5)	0.6426 (9)	0.2966 (4)	0.080 (2)	
H19A	0.4647	0.7008	0.3358	0.120*	
H19B	0.4952	0.6183	0.2685	0.120*	
H19C	0.4232	0.5585	0.3145	0.120*	
C20	0.3618 (4)	0.1489 (6)	0.9896 (3)	0.0545 (16)	
H20	0.4026	0.2009	1.0119	0.065*	
C21	0.3507 (6)	0.3432 (7)	0.9072 (4)	0.083 (3)	
H21A	0.3884	0.3854	0.9401	0.125*	
H21B	0.3027	0.4007	0.9034	0.125*	
H21C	0.3761	0.3349	0.8613	0.125*	
C22	0.2656 (5)	0.1276 (8)	0.8942 (4)	0.076 (2)	
H22A	0.2694	0.0294	0.9052	0.113*	
H22B	0.2734	0.1412	0.8440	0.113*	
H22C	0.2126	0.1618	0.9077	0.113*	
H1	0.115 (4)	−0.456 (7)	0.222 (3)	0.050 (16)*	

### Atomic displacement parameters (Å^2^)

**Table:**

	*U*^11^	*U*^22^	*U*^33^	*U*^12^	*U*^13^	*U*^23^
Ni1	0.0388 (3)	0.0225 (3)	0.0473 (3)	−0.0047 (2)	−0.0047 (3)	0.0025 (2)
N1	0.036 (2)	0.032 (2)	0.044 (3)	−0.0005 (18)	−0.006 (2)	0.005 (2)
N2	0.040 (3)	0.027 (2)	0.055 (3)	−0.007 (2)	−0.001 (2)	−0.0057 (19)
N3	0.039 (2)	0.0246 (19)	0.046 (2)	−0.0044 (18)	−0.004 (2)	−0.001 (2)
N4	0.040 (3)	0.030 (2)	0.048 (3)	−0.010 (2)	0.002 (2)	−0.0043 (19)
N5	0.050 (3)	0.040 (3)	0.059 (3)	−0.004 (2)	0.007 (3)	−0.009 (2)
N6	0.051 (3)	0.043 (3)	0.057 (3)	−0.003 (2)	0.001 (3)	0.004 (2)
O1	0.052 (2)	0.030 (2)	0.058 (2)	−0.0088 (18)	−0.014 (2)	0.0028 (19)
O2	0.065 (3)	0.045 (2)	0.056 (2)	0.000 (2)	−0.020 (2)	0.0156 (19)
O3	0.067 (3)	0.039 (2)	0.066 (3)	−0.002 (2)	−0.004 (3)	0.016 (2)
O4	0.054 (3)	0.028 (2)	0.095 (4)	−0.0086 (19)	0.011 (2)	0.006 (2)
O5	0.049 (2)	0.033 (2)	0.059 (2)	−0.0060 (17)	−0.013 (2)	0.0011 (19)
O6	0.062 (3)	0.046 (2)	0.051 (2)	−0.001 (2)	−0.012 (2)	0.0124 (19)
O7	0.068 (3)	0.043 (2)	0.069 (3)	−0.002 (2)	−0.002 (3)	0.020 (2)
O8	0.056 (3)	0.028 (2)	0.086 (3)	−0.0050 (19)	0.002 (2)	0.008 (2)
O9	0.043 (2)	0.053 (3)	0.080 (3)	−0.0041 (19)	−0.005 (2)	−0.016 (2)
O10	0.048 (3)	0.048 (2)	0.088 (3)	−0.0001 (19)	−0.007 (2)	0.023 (2)
O1W	0.049 (2)	0.032 (2)	0.066 (3)	−0.0044 (18)	0.006 (2)	−0.0012 (18)
O2W	0.053 (3)	0.0281 (19)	0.065 (3)	−0.0043 (18)	0.003 (2)	−0.0001 (18)
C1	0.037 (3)	0.029 (3)	0.039 (3)	−0.002 (2)	−0.001 (3)	0.006 (2)
C2	0.033 (3)	0.027 (2)	0.043 (3)	0.001 (2)	0.009 (2)	0.002 (2)
C3	0.041 (3)	0.027 (3)	0.049 (3)	0.000 (2)	−0.003 (3)	0.001 (2)
C4	0.042 (4)	0.036 (3)	0.046 (3)	0.000 (3)	−0.005 (3)	0.003 (3)
C5	0.054 (4)	0.029 (3)	0.058 (4)	0.001 (3)	0.013 (3)	0.006 (3)
C6	0.057 (4)	0.048 (3)	0.062 (4)	−0.010 (3)	−0.014 (3)	0.007 (3)
C7	0.111 (7)	0.105 (7)	0.074 (6)	0.009 (5)	−0.011 (5)	0.011 (5)
C8	0.118 (11)	0.112 (11)	0.090 (9)	0.029 (8)	−0.025 (8)	−0.022 (8)
C8'	0.14 (4)	0.10 (3)	0.10 (3)	0.01 (3)	−0.03 (3)	0.00 (2)
C9	0.035 (3)	0.026 (3)	0.041 (3)	−0.002 (2)	0.004 (2)	−0.003 (2)
C10	0.039 (3)	0.027 (3)	0.044 (3)	0.000 (2)	0.002 (3)	−0.002 (2)
C11	0.042 (4)	0.033 (3)	0.046 (3)	−0.002 (2)	−0.004 (3)	−0.004 (3)
C12	0.043 (3)	0.031 (3)	0.045 (3)	0.004 (3)	0.001 (3)	−0.002 (3)
C13	0.040 (3)	0.033 (3)	0.061 (4)	0.002 (3)	0.010 (3)	0.004 (3)
C14	0.065 (4)	0.042 (3)	0.063 (4)	−0.009 (3)	−0.016 (3)	0.010 (3)
C15	0.124 (8)	0.124 (8)	0.077 (6)	0.011 (7)	−0.019 (6)	0.022 (6)
C16	0.13 (3)	0.12 (3)	0.081 (18)	0.00 (2)	−0.03 (2)	0.026 (18)
C16'	0.13 (3)	0.11 (2)	0.081 (14)	−0.005 (15)	−0.005 (16)	0.001 (13)
C17	0.041 (3)	0.041 (3)	0.066 (4)	0.005 (3)	0.001 (3)	−0.001 (3)
C18	0.119 (7)	0.047 (4)	0.084 (5)	0.002 (4)	0.009 (5)	−0.012 (4)
C19	0.071 (5)	0.092 (6)	0.078 (5)	0.013 (5)	0.000 (5)	−0.021 (4)
C20	0.039 (3)	0.057 (4)	0.068 (4)	0.003 (3)	−0.006 (3)	0.001 (3)
C21	0.119 (8)	0.051 (4)	0.081 (5)	−0.008 (4)	0.009 (5)	0.018 (4)
C22	0.080 (5)	0.093 (5)	0.054 (4)	−0.027 (4)	−0.014 (4)	0.021 (4)

### Geometric parameters (Å, °)

**Table:**

Ni1—N1	2.059 (4)	C7—C8'	1.51 (4)
Ni1—N3	2.059 (4)	C7—H7A	0.9700
Ni1—O1W	2.079 (4)	C7—H7B	0.9700
Ni1—O2W	2.080 (4)	C7—H7'A	0.9700
Ni1—O1	2.104 (4)	C7—H7'B	0.9700
Ni1—O5	2.109 (4)	C8—H7'B	1.1455
N1—C3	1.321 (7)	C8—H8A	0.9600
N1—C1	1.379 (6)	C8—H8B	0.9600
N2—C3	1.349 (7)	C8—H8C	0.9600
N2—C2	1.357 (7)	C8'—H8'1	0.9600
N2—H2	0.8600	C8'—H8'2	0.9600
N3—C11	1.331 (7)	C8'—H8'3	0.9600
N3—C9	1.371 (6)	C9—C10	1.378 (7)
N4—C11	1.345 (6)	C9—C12	1.471 (8)
N4—C10	1.391 (7)	C10—C13	1.467 (7)
N4—H4	0.8600	C11—C14	1.468 (8)
N5—C17	1.321 (8)	C14—C15	1.555 (11)
N5—C19	1.420 (9)	C14—H14A	0.9700
N5—C18	1.454 (8)	C14—H14B	0.9700
N6—C20	1.307 (7)	C15—C16'	1.51 (3)
N6—C22	1.443 (8)	C15—C16	1.52 (4)
N6—C21	1.466 (8)	C15—H15A	0.9700
O1—C4	1.259 (6)	C15—H15B	0.9700
O2—C4	1.265 (6)	C15—H15C	0.9700
O3—C5	1.300 (7)	C15—H15D	0.9700
O3—H1	1.05 (6)	C16—H16A	0.9600
O4—C5	1.226 (7)	C16—H16B	0.9600
O5—C12	1.258 (6)	C16—H16C	0.9600
O6—C12	1.263 (6)	C16'—H16D	0.9600
O7—C13	1.281 (7)	C16'—H16E	0.9600
O7—H7	0.8200	C16'—H16F	0.9600
O8—C13	1.229 (7)	C17—H17	0.9300
O9—C17	1.228 (6)	C18—H18A	0.9600
O10—C20	1.230 (7)	C18—H18B	0.9600
O1W—H1W	0.8500	C18—H18C	0.9600
O1W—H2W	0.8500	C19—H19A	0.9600
O2W—H3W	0.8501	C19—H19B	0.9600
O2W—H4W	0.8500	C19—H19C	0.9600
C1—C2	1.370 (7)	C20—H20	0.9300
C1—C4	1.474 (8)	C21—H21A	0.9600
C2—C5	1.471 (7)	C21—H21B	0.9600
C3—C6	1.487 (7)	C21—H21C	0.9600
C6—C7	1.555 (10)	C22—H22A	0.9600
C6—H6A	0.9700	C22—H22B	0.9600
C6—H6B	0.9700	C22—H22C	0.9600
C7—C8	1.463 (14)		
			
N1—Ni1—N3	179.6 (2)	C7—C8—H8C	109.5
N1—Ni1—O1W	88.90 (16)	C7—C8'—H8'1	109.5
N3—Ni1—O1W	91.00 (16)	C7—C8'—H8'2	109.5
N1—Ni1—O2W	90.96 (16)	H8'1—C8'—H8'2	109.5
N3—Ni1—O2W	89.13 (16)	C7—C8'—H8'3	109.5
O1W—Ni1—O2W	179.68 (18)	H8'1—C8'—H8'3	109.5
N1—Ni1—O1	80.44 (15)	H8'2—C8'—H8'3	109.5
N3—Ni1—O1	99.99 (16)	N3—C9—C10	110.0 (5)
O1W—Ni1—O1	88.97 (14)	N3—C9—C12	118.2 (4)
O2W—Ni1—O1	90.72 (16)	C10—C9—C12	131.7 (5)
N1—Ni1—O5	99.23 (15)	C9—C10—N4	104.4 (4)
N3—Ni1—O5	80.33 (15)	C9—C10—C13	132.8 (5)
O1W—Ni1—O5	90.31 (16)	N4—C10—C13	122.6 (5)
O2W—Ni1—O5	90.00 (15)	N3—C11—N4	110.2 (5)
O1—Ni1—O5	179.21 (17)	N3—C11—C14	126.3 (5)
C3—N1—C1	106.1 (4)	N4—C11—C14	123.4 (5)
C3—N1—Ni1	143.2 (4)	O5—C12—O6	124.2 (5)
C1—N1—Ni1	110.7 (3)	O5—C12—C9	116.8 (5)
C3—N2—C2	108.9 (4)	O6—C12—C9	119.0 (5)
C3—N2—H2	125.6	O8—C13—O7	124.1 (5)
C2—N2—H2	125.6	O8—C13—C10	119.8 (5)
C11—N3—C9	106.7 (4)	O7—C13—C10	116.1 (5)
C11—N3—Ni1	142.7 (4)	C11—C14—C15	111.1 (5)
C9—N3—Ni1	110.6 (3)	C11—C14—H14A	109.4
C11—N4—C10	108.7 (4)	C15—C14—H14A	109.4
C11—N4—H4	125.6	C11—C14—H14B	109.4
C10—N4—H4	125.6	C15—C14—H14B	109.4
C17—N5—C19	122.1 (5)	H14A—C14—H14B	108.0
C17—N5—C18	119.5 (6)	C16'—C15—C14	114.0 (14)
C19—N5—C18	118.4 (6)	C16—C15—C14	109.9 (19)
C20—N6—C22	120.8 (5)	C16'—C15—H15A	129.8
C20—N6—C21	121.3 (6)	C16—C15—H15A	109.7
C22—N6—C21	117.9 (6)	C14—C15—H15A	109.7
C4—O1—Ni1	114.2 (3)	C16—C15—H15B	109.7
C4—O2—H1	111 (2)	C14—C15—H15B	109.7
C5—O3—H1	113 (3)	H15A—C15—H15B	108.2
C12—O5—Ni1	114.0 (4)	C16'—C15—H15C	98.8
C13—O7—H7	109.5	C14—C15—H15C	107.5
Ni1—O1W—H1W	112.5	C16'—C15—H15D	119.5
Ni1—O1W—H2W	125.5	C14—C15—H15D	108.6
H1W—O1W—H2W	109.5	H15C—C15—H15D	107.2
Ni1—O2W—H3W	105.4	C15—C16—H16A	109.5
Ni1—O2W—H4W	106.7	C15—C16—H16B	109.5
H3W—O2W—H4W	108.3	C15—C16—H16C	109.5
C2—C1—N1	109.5 (5)	C15—C16'—H16D	109.5
C2—C1—C4	132.8 (5)	C15—C16'—H16E	109.5
N1—C1—C4	117.7 (4)	H16D—C16'—H16E	109.5
N2—C2—C1	105.4 (4)	C15—C16'—H16F	109.5
N2—C2—C5	122.6 (5)	H16D—C16'—H16F	109.5
C1—C2—C5	131.9 (5)	H16E—C16'—H16F	109.5
N1—C3—N2	110.2 (5)	O9—C17—N5	124.7 (6)
N1—C3—C6	124.9 (5)	O9—C17—H17	117.7
N2—C3—C6	124.9 (5)	N5—C17—H17	117.7
O1—C4—O2	124.6 (5)	N5—C18—H18A	109.5
O1—C4—C1	116.9 (5)	N5—C18—H18B	109.5
O2—C4—C1	118.4 (5)	H18A—C18—H18B	109.5
O4—C5—O3	123.9 (5)	N5—C18—H18C	109.5
O4—C5—C2	119.6 (6)	H18A—C18—H18C	109.5
O3—C5—C2	116.5 (5)	H18B—C18—H18C	109.5
C3—C6—C7	112.9 (6)	N5—C19—H19A	109.5
C3—C6—H6A	109.0	N5—C19—H19B	109.5
C7—C6—H6A	109.0	H19A—C19—H19B	109.5
C3—C6—H6B	109.0	N5—C19—H19C	109.5
C7—C6—H6B	109.0	H19A—C19—H19C	109.5
H6A—C6—H6B	107.8	H19B—C19—H19C	109.5
C8—C7—C6	114.1 (9)	O10—C20—N6	125.2 (6)
C8'—C7—C6	130.5 (19)	O10—C20—H20	117.4
C8—C7—H7A	108.7	N6—C20—H20	117.4
C6—C7—H7A	108.7	N6—C21—H21A	109.5
C8—C7—H7B	108.7	N6—C21—H21B	109.5
C8'—C7—H7B	120.0	H21A—C21—H21B	109.5
C6—C7—H7B	108.7	N6—C21—H21C	109.5
H7A—C7—H7B	107.6	H21A—C21—H21C	109.5
C8'—C7—H7'A	106.8	H21B—C21—H21C	109.5
C6—C7—H7'A	104.3	N6—C22—H22A	109.5
C8'—C7—H7'B	103.8	N6—C22—H22B	109.5
C6—C7—H7'B	104.0	H22A—C22—H22B	109.5
H7A—C7—H7'B	147.0	N6—C22—H22C	109.5
H7'A—C7—H7'B	105.4	H22A—C22—H22C	109.5
C7—C8—H8A	109.5	H22B—C22—H22C	109.5
C7—C8—H8B	109.5		

### Hydrogen-bond geometry (Å, °)

**Table:**

*D*—H···*A*	*D*—H	H···*A*	*D*···*A*	*D*—H···*A*
O3—H1···O2	1.05 (6)	1.42 (6)	2.474 (6)	176 (5)
O7—H7···O6	0.82	1.67	2.479 (6)	169
N2—H2···O10^i^	0.86	1.93	2.780 (6)	171
N4—H4···O9	0.86	1.94	2.788 (6)	171
O1W—H1W···O8^ii^	0.85	1.96	2.782 (5)	162
O1W—H2W···O10^iii^	0.85	1.92	2.757 (6)	168
O2W—H3W···O4^iv^	0.85	1.96	2.794 (5)	166
O2W—H4W···O9^v^	0.85	1.98	2.800 (6)	163

Symmetry codes: (i) *x*−1/2, −*y*−1/2, *z*−1; (ii) *x*, *y*−1, *z*; (iii) *x*, *y*, *z*−1; (iv) *x*, *y*+1, *z*; (v) *x*−1/2, −*y*+1/2, *z*.
